# Proteomic analysis of *Potentilla fruticosa* L. leaves by iTRAQ reveals responses to heat stress

**DOI:** 10.1371/journal.pone.0182917

**Published:** 2017-08-22

**Authors:** Yingtian Guo, Zhi Wang, Xuelian Guan, Zenghui Hu, Ze Zhang, Jian Zheng, Yizeng Lu

**Affiliations:** 1 College of Landscape Architecture, Beijing University of Agriculture, Beijing, China; 2 Key Laboratory of Plant Molecular Physiology, Institute of Botany, Chinese Academy of Sciences, Beijing, China; 3 Beijing Collaborative Innovation Center for Eco-Environmental Improvement with Forestry and Fruit Trees, Beijing, China; 4 Shandong Forest Germplasm Resources Center, Jinan City, Shandong Province, China; Universidade de Lisboa Instituto Superior de Agronomia, PORTUGAL

## Abstract

High temperature is an important environmental factor that affects plant growth and crop yield. *Potentilla fruticosa* L. has a developed root system and characteristics of resistance to several stresses (e.g., high temperature, cold, drought) that are shared by native shrubs in the north and west of China. To investigate thermotolerance mechanisms in *P*. *fruticosa*, 3-year-old plants were subjected to a high temperature of 42°C for 1, 2, and 3 days respectively before analysis. Then, we studied changes in cell ultrastructure using electron microscopy and investigated physiological changes in the leaves of *P*. *fruticosa*. Additionally, we used isobaric tags for relative and absolute quantification (iTRAQ) coupled with liquid chromatography-tandem mass spectrometry (LC-MS/MS) to study proteomic changes in *P*. *fruticosa* leaves after 3 d of 42°C heat stress. we found that the cell membrane and structure of chloroplasts, especially the thylakoids in *P*. *fruticosa* leaves, was destroyed by a high temperature stress, which might affect the photosynthesis in this species. We identified 35 up-regulated and 23 down-regulated proteins after the heat treatment. Gene Ontology (GO) analysis indicated that these 58 differentially abundant proteins were involved mainly in protein synthesis, protein folding and degradation, abiotic stress defense, photosynthesis, RNA process, signal transduction, and other functions. The 58 proteins fell into different categories based on their subcellular localization mainly in the chloroplast envelope, cytoplasm, nucleus, cytosol, chloroplast, mitochondrion and cell membrane. Five proteins were selected for analysis at the mRNA level; this analysis showed that gene transcription levels were not completely consistent with protein abundance. These results provide valuable information for *Potentilla* thermotolerance breeding.

## Introduction

Plant growth and development are affected by a variety of biotic stress factors, such as bacterial infection, symbiotic or parasitic microbial infection, and abiotic stress factors, such as drought, flood, salinization, heat, cold, and mechanical damage. Adverse effects of stress on plants lead to a series of physiological changes in metabolic and biochemical processes that cause irreversible damage to growth and development [1, 2] and can result in plant death. Of these various stress factors, high temperature damage to plants is particularly important and affects plant growth and crop yields [1–3]. When exposed to a high temperature stress, plants generally respond through changes in cell structure, cell membrane permeability, cell osmotic adjustment, and photosynthetic activity [4]. These stress responses can be examined by elucidating the changes in protein content (proteome) of cells. Using proteomics, the effects of stress on protein abundance have been examined in the model dicot *Arabidopsis thaliana* [5–7], horticultural plants such as *Cucumis sativus*, *Solanum tuberosum* [8], and *Vitis vinifera* [9], crop species such as *Oryza sativa* [10], *Zea mays* [11] and *Glycine max* [12], and model tree species such as *Picea asperata* [13] and *Populus euphratica* [14]. The analyses of the changes in protein abundance in response to different stresses have identified the metabolic pathways, stress response signals, signal transduction pathways, and self-repair mechanisms that are affected in all these plant species. Characterization of the factors involved in stress response provides valuable information for use in resistance breeding in high-quality plant species.

*Potentilla fruticosa* L. is a long-flowering, deciduous shrub found in the highland forest of northern China. The root system of the plant is developed and shows resistance to cold and drought; this resistance enables the plant to tolerate temperatures below -30°C and above 39°C. It can also survive on infertile soil, and shows resistance to many pests and diseases. *P*. *fruticosa* has been termed the "King of flower life" and has been used as a hedge or ornamental plant; these properties make it an important commercial agricultural product. To date, however, there have been comparatively few studies on *P*. *fruticosa* although research into propagation techniques [15, 16], feeding value [17, 18], and community characteristics [19, 20] have been performed.

In this study, we sought to determine the nature of the proteomic changes that occur in *P*. *fruticosa* after exposure to heat stress. We observed and analyzed the changes to ultrastructure and physiology in *P*. *fruticosa* leaves exposed to different durations of high temperature stress. At the same time, we used the isobaric tags for relative and absolute quantitation (iTRAQ) method to compare the abundances of different proteins under normal and heat-stressed conditions. The obtained data will provide an important bioinformatic resource for investigating response mechanisms in *P*. *fruticosa* to thermal stress.

## Materials and methods

### Plant material and temperature treatment

Eight, three-year-old *P*. *fruticosa* plants were randomly selected from the forestry resource nursery at Beijing Agricultural University, a resource conservation unit of the National Forest Genetic Resources Platform (NFGRP) in Beijing, China. All the temperature treatments were carried out under controlled environmental conditions in an artificial climate chamber (PGX-350D; Ningbo Saifu, China). The plants were pretreated under standard conditions (photoperiod 14 h light/10 h dark, day/night temperature 30/20°C, 70% relative humidity, light intensity 300 μmol • m^-2^ • s^-1^) for 3 d; then, mature, fully expanded leaves were collected as a control. The day temperature was immediately adjusted to 42°C after harvesting control leaves, while the other conditions remained unchanged. The plants were subjected to 42/20°C (day/night) for 1, 2, or 3 d, and leaves were collected from each time point as treatment groups. For the iTRAQ analysis, two biological replicate experiments (a total of 16 plants) were performed; for the other analyses, three replicates (a total of 24 plants) were used.

### Analysis of mesophyll cell ultrastructure

Leaves were cut into small pieces (approximately 1 mm^2^ and avoiding the main vein) and fixed in 3% glutaraldehyde for 4 h at room temperature. After washing in 0.1 M phosphate buffer (pH 7.1), the leaf samples were post-fixed in 1% osmium tetroxide for 3 h and then dehydrated in cold ethanol. The samples were embedded in Epon-618 polymerization resin for 12 h at 37°C, which was hardened by 24 h at 45°C, followed by 48 h at 60°C. The embedding block was trimmed and 70 nm thick sections were cut using a Leica EM UC7 microtome (Leica, Germany). The sections were stained with uranyl acetate and lead citrate, and analyzed using a Hitachi-7500 transmission electron microscope (Hitachi, Japan).

### Physiological parameter measurements

Leaves were washed with deionized water and leaf discs were cut (8 mm diameter). The method of Qingsheng [21] was used to measure relative electrolytic leakage (REL). Malondialdehyde (MDA) contents of leaves were determined using commercial assay kits purchased from Nanjing Jiancheng Bioengineering Institute (Nanjing, China), Fresh leaves (0.1 g) were ground to powder in liquid nitrogen and suspended in 0.9 mL phosphate buffer (0.1 M, pH 7.0–7.4). The suspensions were centrifuged at 3,500 × *g* for 10 min at 4°C, and supernatants were collected for MDA measurements using the manufacturer’s suggested protocol.

### Protein extraction

Each sample from the control and heat-treated plants was dissolved in lysis buffer (7 M urea, 2 M thiourea, 0.1% CHAPS), lysed by sonication, and then extracted at room temperature (25°C) for 30 min and centrifuged at 15,000 × *g* for 20 min at 4°C. The supernatant was collected, stored at -80°C, and subsequently quantified by the Bradford method [22].

### Protein digestion and iTRAQ labeling

A total of 200 μl of protein solution was placed in a centrifuge tube with 4 μl Reducing Reagent (AB Sciex, Foster City, CA, USA). Samples were incubated for 1 h at 60°C, and then 2 μl Cysteine-Blocking Reagent was added (AB Sciex) and the mixture was incubated for 10 min at room temperature. After reduction, alkylation, and trypsin-digestion, the samples were labeled with iTRAQ^®^ reagents using the 8-plex kit protocol (AB Sciex). The sample labeling is shown in the Table 1.

**Table 1:** Information of the sample labeling.

Number	Sample	Corresponding isotopes
1	control	113
2	1 day	114
3	2 day	115
4	3 day	116
5	control	117
6	1 day	118
7	2 day	119
8	3 day	121

### Peptide fractionation by reversed-phase high performance liquid chromatography

Using a RIGOL L-3000 HPLC system (RIGOL, Beijing, China) connected to a chromatographic column (Durashell-C18, 4.6 mm × 250 mm, 5 μm, 100 Å; Agela, Tianjing, China), the labeled samples were fractionated under high pH conditions. First, the labeled samples were mixed and dissolved in 100 μl of mobile phase A [98% ddH_2_O, 2% acetonitrile (pH 10)], then centrifuged at 14,000 × *g* for 20 min and the supernatants were collected. Second, 100 μl of sample was added and subjected to a flow rate of 0.7 ml/min of mobile phase B [98% acetonitrile, 2% ddH_2_O (pH 10)]. The following gradient was applied: 0 min, 5% mobile phase B; 5 min, 8% mobile phase B; 35 min, 18% mobile phase B; 62 min, 32% mobile phase B; 64 min, 95% mobile phase B; 68 min, 95% mobile phase B; 72 min, 5% mobile phase B.

### Liquid chromatographic tandem mass spectrometry

The obtained fractions were re-dissolved in 20 μl solution A (100% ultra-pure water, 0.1% formic acid) and the samples were separated by LC-MS using an chromatographic EASY-Spray column (12 cm × 75 μm, C18, 3 μm; Thermo Fisher, Waltham, USA) and a loading pump flow rate of 350 nl/min for 15 min and a separation flow rate of 350 nl/min. The following gradient was applied: 0 min, 4% mobile phase B (100% acetonitrile, 0.1% formic acid); 5 min, 15% mobile phase B; 40 min, 25% mobile phase B; 65 min, 35% mobile phase B; 70 min, 95% mobile phase B; 82 min, 95% mobile phase B; 85 min, 4% mobile phase B; 90 min, 4% mobile phase B. An ABI-5600 (AB SCIEX, USA) mass spectrometer was used for protein analysis with the parameters: 2.1 kv Spray voltage; EASY-Spray Ion Source; 350–1800 m/z Full MS scan range.

### Protein identification and quantification

All the mass spectral data were processed with Protein Pilot supported by the ABI company. The parameters were as follows: trypsin as enzyme, carbamidomethylation as a static modification, iTRAQ 8-plex in N-terminal, oxidation in methionine as a dynamic modification. A false discovery rate (FDR) of all peptide and protein identifications of <1%, ±15 ppm precursor ion mass tolerance, ±20 mmu fragment ion mass tolerance and 2 max missed cleavages were applied. Proteins with a fold change of ≥1.5 or ≤0.666 and results were regarded as statistically significant at p-values less than 0.05.

### Bioinformatic analysis of proteins

Identified proteins were searched against the NCBI *Arabidopsis thaliana* protein database using the DAVID bioinformatics analysis tools (https://david.ncifcrf.gov/). Functional annotations of differentially abundant proteins were performed using Gene Ontology (http://www.geneontology.org); the Kyoto Encyclopedia of Genes and Genomes (KEGG) (http://www.genome.jp/kegg/) was used to predict the biological and functional properties of differentially abundant proteins.

### Quantitative real-time PCR

Total RNA was extracted using the EASYspin Plus Plant RNA Kit (Aidlab Biotech, Beijing, China). cDNA was synthesized in a 20 μl reaction volume using *TransScript*^®^ One-Step gDNA Removal and cDNA Synthesis SuperMix (TransGen Biotech, Beijing, China) according to the manufacturer’s protocol. Quantitative (q)PCR was performed using SYBR Premix Ex TaqTMII (Takara, Dalian, China) on the iQ^™^5 Multicolor Real-Time PCR Detection System (BIO-RAD, USA).

Specific primers were designed based on the ORF (open reading frame) sequence of the corresponding proteins using Primer3web V4.0.0 (S1 Table). All real-time PCR reactions were performed in triplicate using *PfActin* as a reference for gene expression. Relative gene expression was calculated using the 2^-ΔΔCt^ method [23].

### Statistical analysis

The control and heat treatment groups were analyzed for statistical significance of differences between multiple groups using one-way ANOVA followed by Duncan's multiple comparisons test. All calculations were performed using SPSS software (version 17.0; IBM, Armonk, NY, USA). All results are presented as mean ± SD from 3 independent biological replications. Differences were considered statistically significant at a P-value less than 0.05.

## Results

### Leaf morphology changes induced by heat stress

**Fig 1:**
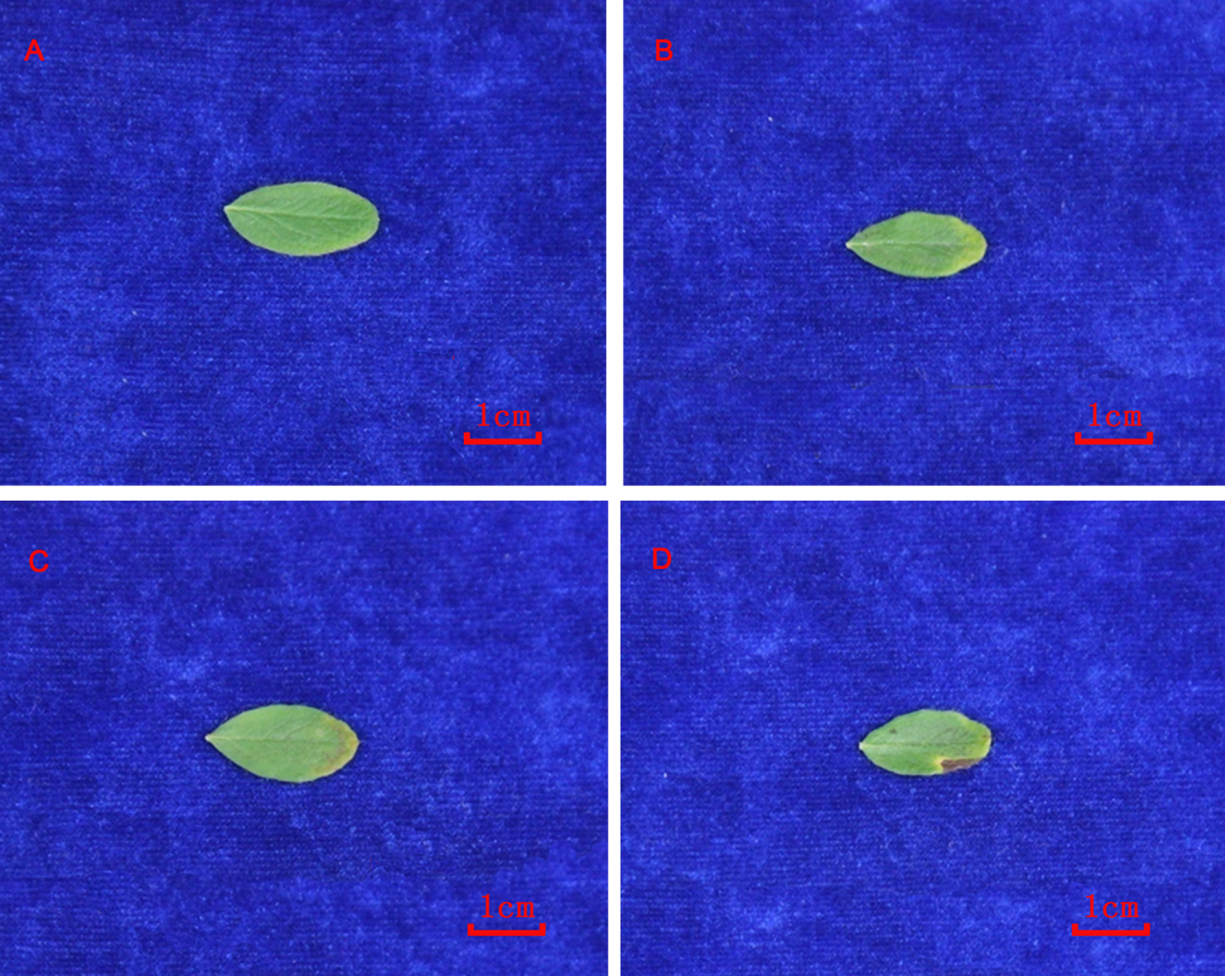
Morphological changes induced by heat stress in *Potentilla fruticosa*. A, B, C, D represent the leaves of control, 1 d, 2 d, and 3 d heat treatment, respectively.

Morphological changes under heat stress in plants can reflect their degree of damage and heat resistance. Here, we found that leaf tips and leaf margins showed evidence of mild heat scorch after 1 d of heat stress (Fig 1B) and leaf margins had obvious evidence of heat scorch after 3 d (Fig 1D) compared to control. This indicated that the degree of leaf injury in *P*. *fruticosa* increased with extended exposure to heat stress.

### Changes to mesophyll cell ultrastructure induced by heat stress

**Fig 2:**
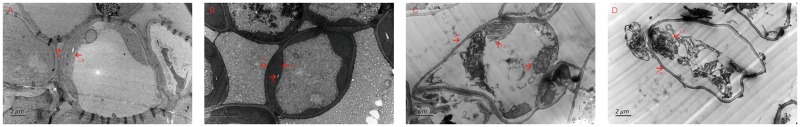
Changes in the mesophyll cell ultrastructure in response to heat stress in *Potentilla fruticosa*. A, B, C, D show the control, 1 d, 2 d, and 3 d heat treatment, respectively. Map scale: 2 μm (A, C, D), 5 μm (B). Ch: chloroplast; Cw: cell wall; Sg: starch grain.

Heat stress can affect the ultrastructure of the plant cell wall, cell membrane, vacuole, chloroplasts, mitochondria, nucleus, and other organelles [24, 25]. In this study, we found that mesophyll cells of *P*. *fruticosa* leaves were deformed under heat stress, and that the cells showed increased distortions on the second and third days of heat stress, indicating that the cell walls and membranes suffered serious injury under the high temperature (Fig 2C and 2D, Panels C and D in S1 Fig). Additionally, chloroplasts enlarged from their normal shuttle-shape on the second and third days of heat stress; the chloroplast thylakoid grana layer disappeared, and the chloroplast appeared to disintegrate. Furthermore, the chloroplast starch granules were fuzzy in appearance, and osmiophilic particles accumulated during the high temperature stress (Panels C and D in S1 Fig). Overall, the cell wall, membrane, chloroplasts, and thylakoid of *P*. *fruticosa* leaf cells suffered serious injury under the high temperature stress.

### Changes in cell membrane permeability during heat stress

**Fig 3:**
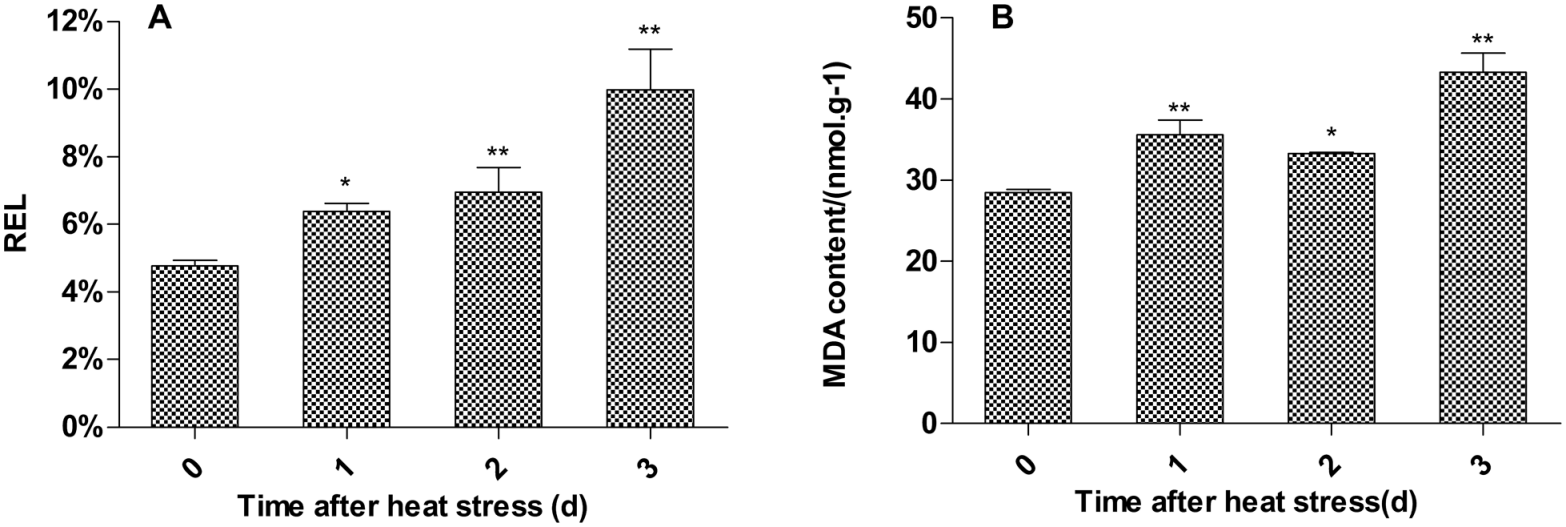
Effect of heat treatment on the relative electrolyte leakage and malondialdehyde (MDA) content in *Potentilla fruticosa* leaves. The horizontal axis shows the control, 1 d, 2 d, and 3 d heat treatment, respectively. Each value represents the mean ± standard deviation (SD) of three replicates. The asterisks indicate the significance of differences the between treatments and their corresponding controls (*P<0.05, **P<0.01).

Under high temperature stress, the structure and function of cell plasma membranes were initially compromised, resulting in an increase in cell membrane permeability and an increased level of the lipid peroxidation product, malondialdehyde (MDA), and intracellular electrolyte leakage. We found that relative electrolyte leakage (REL) and MDA content increased and that there were significant differences between heat stressed plants and controls (Fig 3A and 3B). These findings indicate that the cell membranes of *P*. *fruticosa* leaves suffered damage under the high temperature stress.

### General information on iTRAQ analysis

**Fig 4:**
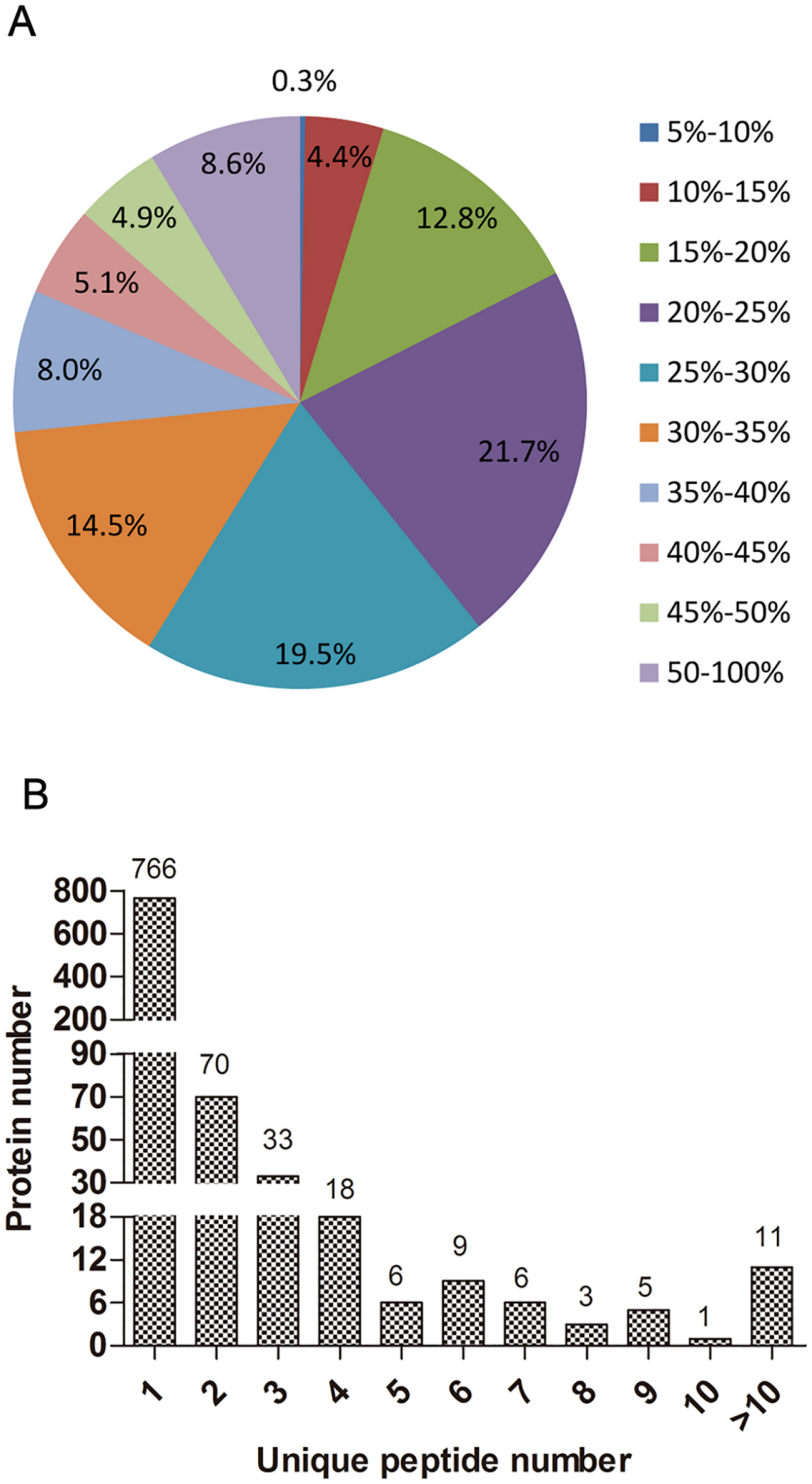
General information on the iTRAQ analysis. (A) Coverage of proteins by using LC-MS/MS-identified peptides. (B) Unique peptides of the detected proteins. The number of proteins in each of the categories is presented above each bar.

Protein and peptide identification was performed using ProteinPilot software. The results for identified proteins and peptides with different false discovery rate (FDR) thresholds are presented in S2 Table. In total, 800 proteins and 1460 peptides were identified with 95% confidence in local FDR. Additionally, 863 proteins and 1577 peptides were identified in global FDR from a fit with threshold of 1%. In total, 995 proteins were identified in the 2 biological replicates. Fig 4 shows the basic information that was generated from the iTRAQ analysis. The distribution analysis of the sequence coverage of the detected peptides indicated that 99.7% of proteins had a coverage of >10% (Fig 4A) and that 17.46% were inferred from at least 2 unique peptides (Fig 4B).

### Identification of differentially abundant proteins

**Fig 5:**
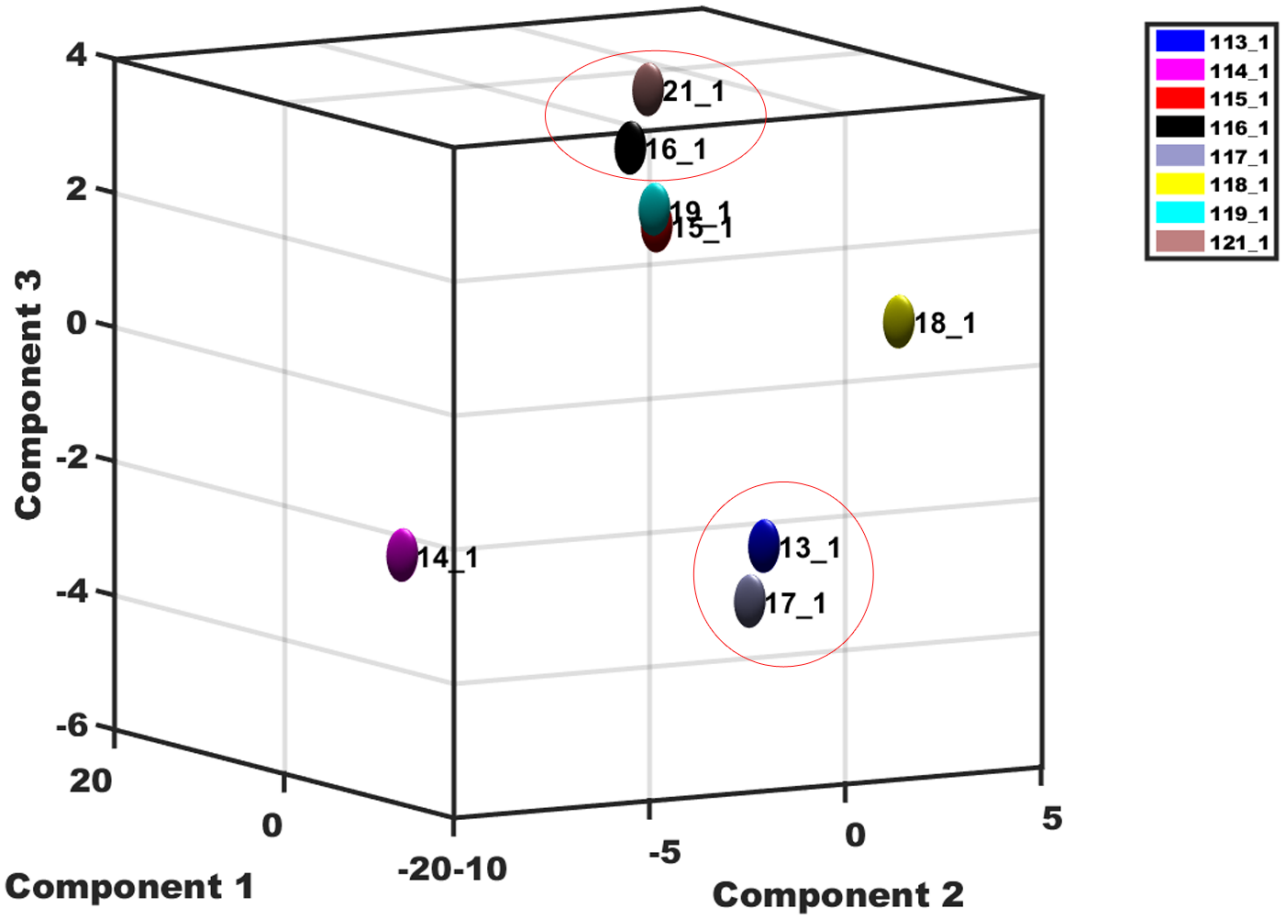
Correlation between the values of the two biological replicates. 113_1/117_1, 114_1/118_1, 115_1/119_1, and 116_1/121_1 represent the leaves in the control and 1 d, 2 d, and 3 d heat treatments, respectively.

We used a principal component analysis (PCA) to examine the correlation relationships between the values of the two biological replicates; this analysis showed a poor correlation for the first day at 42°C (114_1 and 118_1) (Fig 5).Our iTRAQ analysis of the proteome of *P*. *fruticosa* leaves after 42°C treatment for 3 d identified 58 proteins that showed different levels of abundance in the treated plants (P ≤ 0.05) and a fold change of ≥1.5 or ≤0.666; 35 of the proteins were up-regulated and 23 proteins showed a decrease (S3 Table).

### GO function analysis of differentially abundant proteins under heat stress

**Fig 6:**
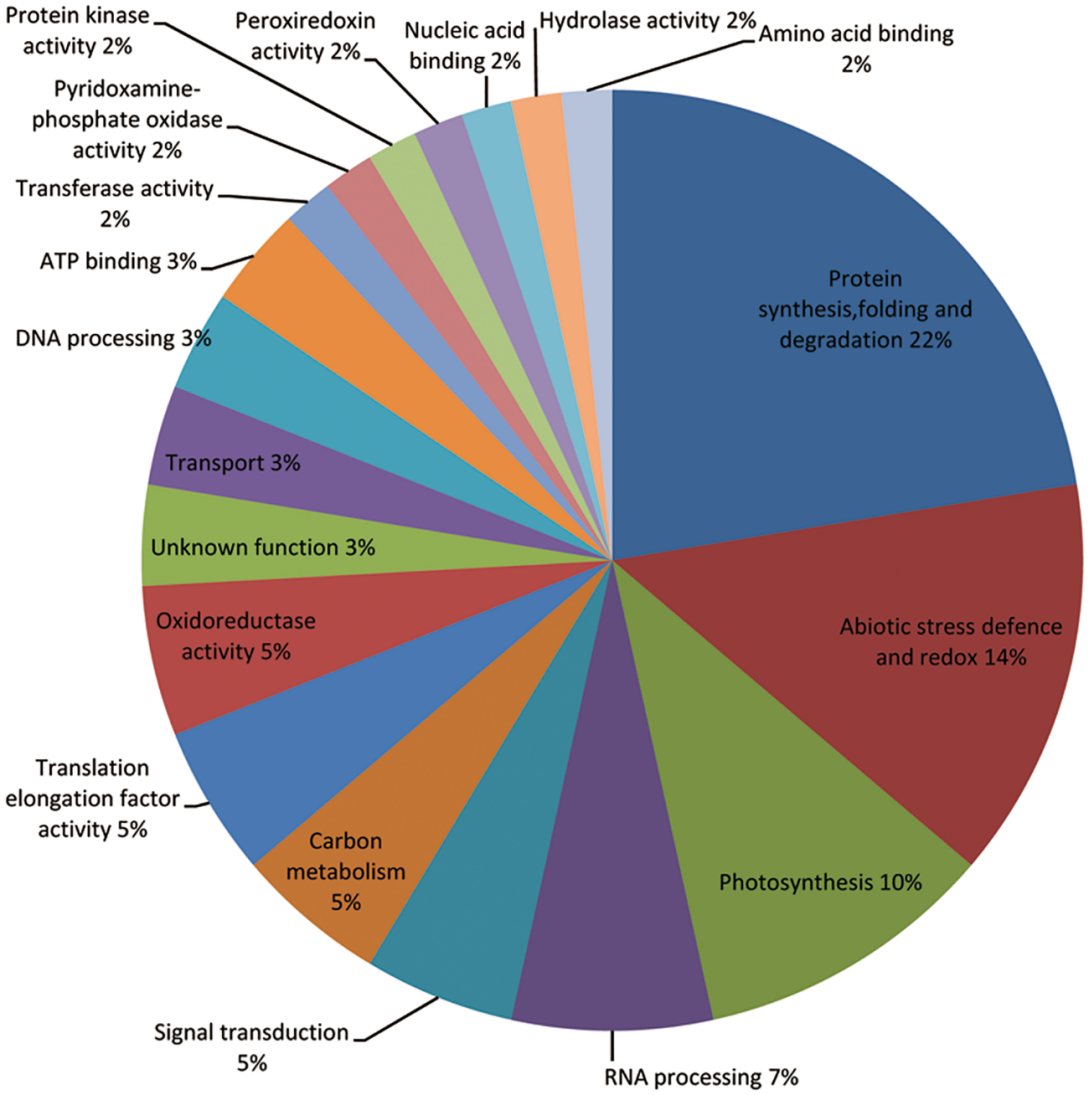
Functional classification of differentially abundant proteins under heat stress.

**Fig 7:**
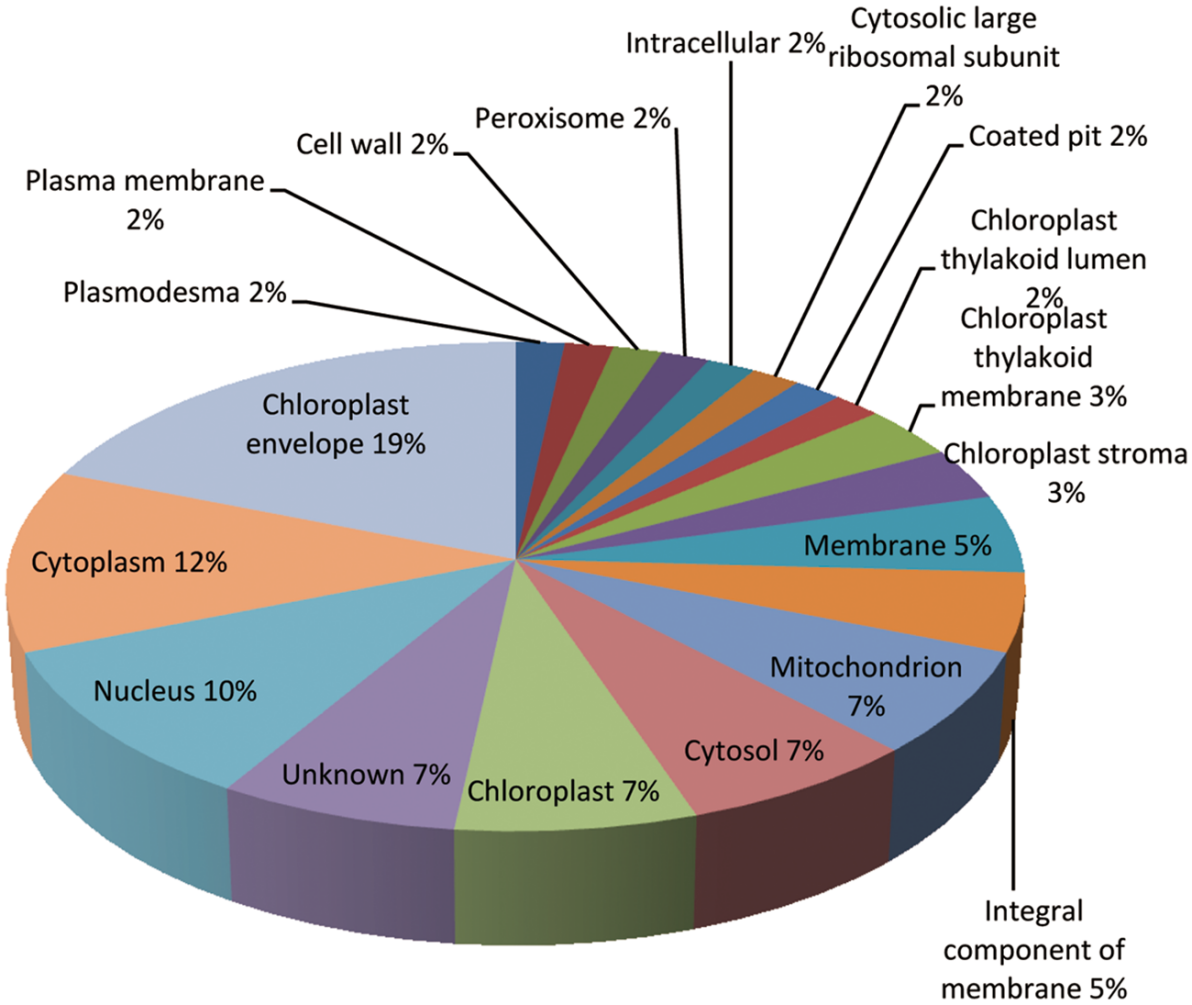
Subcellular localization of the differentially abundant proteins under heat stress.

We used the DAVID bioinformatics tools to align the 58 differentially abundant proteins in the Gene Ontology (GO) database. The 58 differentially abundance proteins had been carried out using three different criteria of protein functional annotation, that is, biological processes, molecular functions class, and cellular components (S4 Table). In addition, the GO enrichment analysis showed that differentially abundant proteins were mainly enriched in chloroplast envelope (GO:0009941), chloroplast thylakoid membrane (GO:0009535) of cellular components and translation elongation factor activity (GO:0003746), poly(U) RNA binding (GO:0008266) of molecular functions and response to heat (GO:0009408), response to hydrogen peroxide (GO:0042542) of biological processes (S5 Table). We also used Blast2GO to classify the functions of the 58 proteins and found that they could be assigned to 18 functional GO categories (Fig 6): 13 proteins (~22%) were involved in protein synthesis, folding, and degradation; eight proteins (~14%) were involved in abiotic stress resistance and oxidation-reduction reaction; and 6 proteins (~10%) were involved in photosynthesis. Some proteins were found to be involved in ATP-binding, transferase activity, and nucleic acid binding. We also analyzed the cellular localization of the 58 proteins (Fig 7). Approximately 19% were located in the chloroplast envelope, 12% in the cytoplasm, 10% in the nucleus, and 7% in the chloroplast, cytosol and mitochondrion, respectively. Others were located in the cell wall, cell membrane, plasmodesmata, and peroxisomes (Fig 7).

### Comparison of mRNA and protein levels of heat-responsive proteins

**Fig 8:**
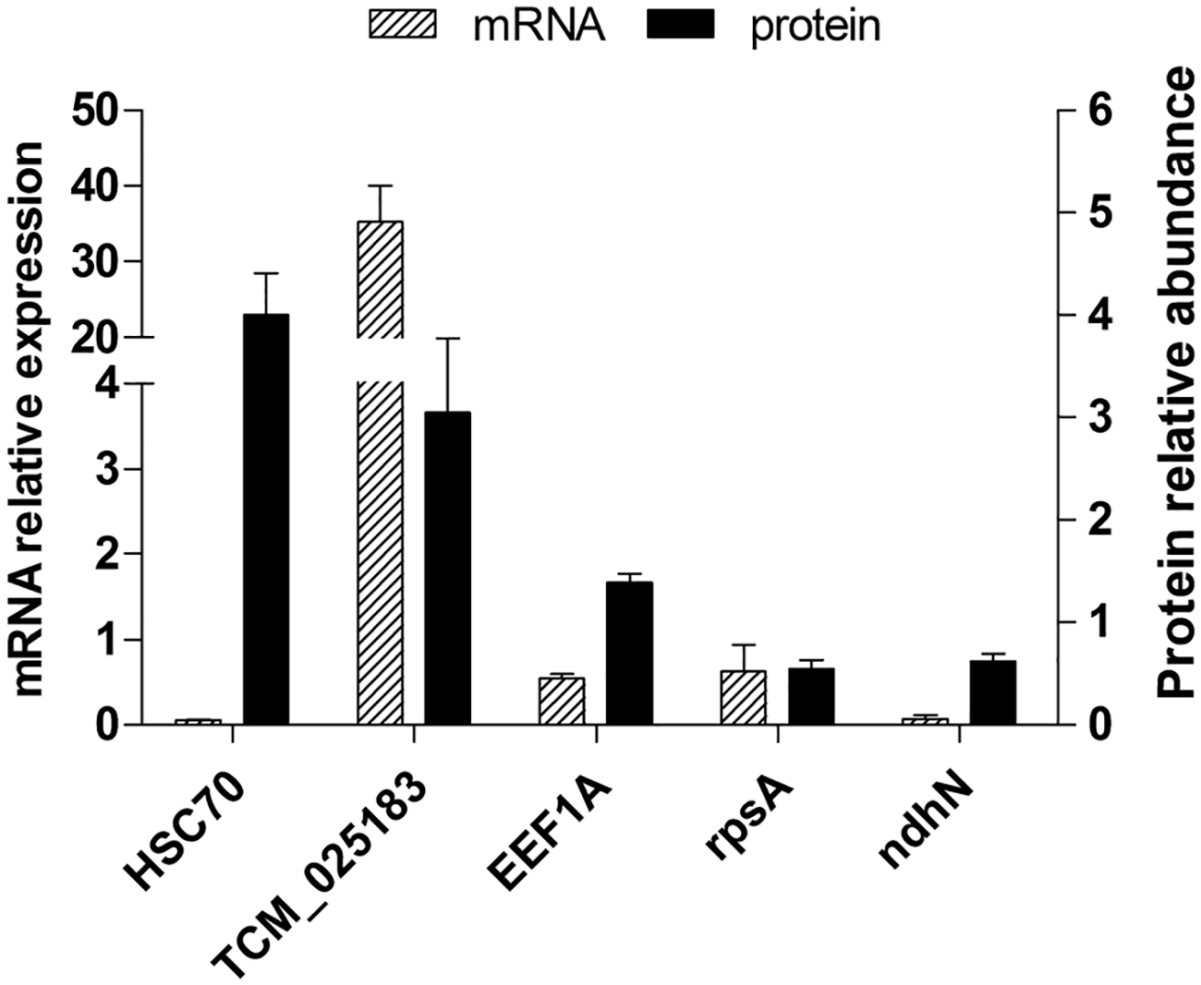
Comparison of the mRNA expression patterns and protein abundance of heat-responsive proteins. HSC70, heat shock cognate 70 kDa protein; TCM_025183, Translation protein SH3-like family protein; EEF1A, Elongation factor 1-alpha-like; rpsA, 30S ribosomal protein S1; ndhN, NAD(P)H-quinone oxidoreductase subunit N-like. The left vertical shows the mRNA relative expression and the right vertical shows the protein relative abundance.

To determine whether the changes in protein abundance identified by iTRAQ correlated with changes at the gene transcription level, we performed qPCR analysis. Five of the 58 proteins were selected for analysis, namely, heat shock cognate 70 kDa protein (HSC70), translation protein SH3-like family protein, elongation factor 1-alpha-like, 30S ribosomal protein S1, and NAD(P)H-quinone oxidoreductase subunit N-like. As shown in Fig 8, the mRNA expression levels of the translation protein SH3-like family protein, 30S ribosomal protein S1 and NAD(P)H-quinone oxidoreductase subunit N-like genes were positively correlated with their observed protein levels. However, HSC70 and the elongation factor 1-alpha-like showed different gene expression patterns in comparison to their protein abundances. The mRNA levels of *HSC70* and *elongation factor 1-alpha-like* were down-regulated whereas the protein levels were increased. These results also support previous reports that transcription patterns are not always directly correlated with protein levels [26–28]. This effect might be due to the time shift between the detection of gene transcription and translation of proteins [26].

## Discussion

### Effects of heat stress on morphological and physiological parameters in *Potentilla fruticosa*

The metabolic, physiological and biochemical processes of plants under normal conditions are relatively stable and coordinated, but environmental stresses can cause changes to metabolic activities [29]. Plant responses to heat stress involve changes to cell structure, cell membrane permeability, and photosynthesis physiology. In this study, we measured the relative conductivity and MDA content of *P*. *fruticosa* leaves exposed to a heat treatment. We found that both of these increased (Fig 2), indicating that cell membrane injury had occurred under the heat stress conditions.

Photosynthesis plays an important role in biosynthesis and energy metabolism in plants, and is very sensitive to temperature changes. Chloroplasts are the site of photosynthesis and have a level of structural stability that is closely related to heat resistance [30]. In this study, we found that the structure of chloroplasts, especially the thylakoids in *P*. *fruticosa* leaves, was destroyed by a high temperature stress (Fig 2). The high temperature stress destroyed chloroplast structure and affected photosynthesis in the plants. To provide a more comprehensive description of the effects of heat stress on cell function, we performed an iTRAQ analysis to measure changes in protein abundance.

### Proteins involved in synthesis, folding and degradation are altered by heat stress

During the heat stress treatment, a large number of proteins associated with protein synthesis, folding, and degradation were either significantly increased or decreased. Elongation factor, which is in a class of protein synthesis factors, catalyzes amino acid extension on the ribosome and controls protein synthesis. The elongation factors in eukaryotes have been identified as eEF-1 and eEF-2. The eEF-1 factor includes four subunits: EF-1α, EF-1β', EF-1β and EF-1γ, and mediate the binding of ammonia acyl-tRNA and ribosomes [31, 32]. eEF-1α is a major translation factor that plays an important role in the control of protein synthesis. The expression of the *EF1-α* gene can be induced by abiotic stresses, such as drought, cold, and heat shock [32]. Bukovnik et al. [33] found that heat stress could induce accumulation of EF-1α in mature plants of spring wheat and that EF-1α was important to wheat response to heat stress. EF-1γ acts as a catalyst in the synthesis of proteins and may be involved in guiding protein synthesis related to cellular structure [34]. In this study, we found that the levels of EF-1α and EF-1γ proteins were increased, indicating that EF-1 might be involved in heat stress resistance in *P*. *fruticosa* by influencing the stability of cellular structures (Table 2). Ribosomal proteins are required for the synthesis of ribosomes and proteins [35], and play a key role in cell metabolism, cell division and cell growth [36]. In this study, we found the levels of two ribosomal proteins (the 30S ribosomal protein S1 and 60S ribosomal protein L13a) were reduced after heat treatment, showing that the synthesis of some proteins was down-regulated by heat stress (Table 2).

**Table 2:** Selected differentially abundant proteins in *Potentilla fruticosa* leaves.

Accession	Unused	%Cov (95)^a^	Peptides (95%)^b^	Protein name	*Species*	Fold change (Heat vs Control)
**Protein synthesis, folding and degradation**						
gi|470126953	5.12	4.93	1	Heat shock 22 kDa protein	*Fragaria vesca* subsp. *vesca*	27.29
gi|470126676	9.71	13.95	5	Chaperone protein ClpB1-like	*Fragaria vesca* subsp. *vesca*	16.904
gi|470129154	17.82	22.57	15	Luminal-binding protein 5-like	*Fragaria vesca* subsp. *vesca*	4.932
gi|470101899	11.75	23.8	13	Heat shock cognate 70 kDa protein-like	*Fragaria vesca* subsp. *vesca*	3.996
gi|470136963	14.4	34.44	9	Chaperone protein ClpB3	*Fragaria vesca* subsp. *vesca*	24.262
gi|470135104	2.67	7.94	1	26.5 kDa heat shock protein	*Fragaria vesca* subsp. *vesca*	13.332
gi|470116855	10.33	37.23	9	18.2 kDa class I heat shock protein-like	*Fragaria vesca* subsp. *vesca*	29.651
gi|470137715	12.37	18.68	6	Peptidyl-prolyl cis-trans isomerase CYP38	*Fragaria vesca* subsp. *vesca*	0.387
gi|659134080	8.33	34	4	Heat shock protein 90–2, partial	*Cucumis melo*	8.486
gi|470147843	7.01	4.04	2	Calnexin homolog 1-like	*Fragaria vesca* subsp. *vesca*	3.771
gi|470122167	5.39	11.48	2	60S ribosomal protein L13a-4-like	*Fragaria vesca* subsp. *vesca*	0.331
gi|590638131	5.82	18.71	2	Translation protein SH3-like family protein	*Theobroma cacao*	3.911
gi|694380111	21.12	59.84	19	Heat shock 70 kDa protein	*Pyrus × bretschneideri*	15.955
gi|470121863	0.26	0	1	REVERSED: uncharacterized protein LOC101301382	*Fragaria vesca* subsp. *vesca*	0.649
gi|645235988	22.89	22.82	17	Elongation factor 1-alpha-like	*Prunus mume*	1.556
gi|225465198	20.16	27.47	8	Elongation factor 1-gamma-like	*Vitis vinifera*	1.706
gi|470117806	6.44	35.59	4	30S ribosomal protein S1	*Fragaria vesca* subsp. *vesca*	0.466
**Abiotic stress defense and redox**						
gi|470110102	10.28	21.88	5	Thioredoxin-like protein CDSP32	*Fragaria vesca* subsp. *vesca*	0.479
gi|470121124	7.98	10.62	3	Glutathione reductase	*Fragaria vesca* subsp. *vesca*	1.941
gi|470104065	2.85	6.62	1	Uncharacterized protein LOC101298064	*Fragaria vesca* subsp. *vesca*	3.192
gi|470121591	6.22	22.62	4	Translationally-controlled tumor protein homolog	*Fragaria vesca* subsp. *vesca*	4.04
gi|470120512	17.69	18.73	7	Linoleate 13S-lipoxygenase 2–1	*Fragaria vesca* subsp. *vesca*	0.069
gi|470121668	3.03	6.75	1	Major allergen Pru ar 1-like	*Fragaria vesca* subsp. *vesca*	0.39
gi|470133785	5.95	10.53	4	Uncharacterized protein At2g37660	*Fragaria vesca* subsp. *vesca*	1.854
gi|470127015	0.17	0	1	Cytochrome P450 716B2-like	*Fragaria vesca* subsp. *vesca*	18.727
gi|470102448	10.74	32.9	7	Peroxiredoxin Q	*Fragaria vesca* subsp. *vesca*	0.27
gi|694452607	0.11	0	1	Beta-amyrin 28-oxidase-like	*Pyrus × bretschneideri*	0.337
gi|470117411	5.79	18.01	3	NAD(P)H-quinone oxidoreductase subunit N-like	*Fragaria vesca* subsp. *vesca*	0.207
gi|470136232	9.87	16.3	5	Uncharacterized oxidoreductase At1g06690	*Fragaria vesca* subsp. *vesca*	0.597
**Photosynthesis**						
gi|470102732	29.3	37.36	15	Ferredoxin—NADP reductase	*Fragaria vesca* subsp. *vesca*	0.259
gi|470108717	18.23	22.83	8	Chloroplast stem-loop binding protein of 41 kDa a	*Fragaria vesca* subsp. *vesca*	0.495
gi|470124625	0.17	0	1	Protein CHUP1	*Fragaria vesca* subsp. *vesca*	0.175
gi|428697270	19.48	40.17	17	ATP synthase CF1 alpha subunit	*Fragaria virginiana*	0.231
gi|595817691	5.48	5.26	2	Hypothetical protein PRUPE_ppa002248mg	*Prunus persica*	1.629
gi|470116673	4.01	13.38	2	Pheophorbide a oxygenase, chloroplastic-like	*Fragaria vesca* subsp. *vesca*	7.673
gi|470108684	4.36	8.46	2	37 kDa inner envelope membrane protein	*Fragaria vesca* subsp. *vesca*	0.325
**RNA metabolism**						
gi|470133931	0.45	0	1	Serine/arginine-rich splicing factor RS2Z33-like	*Fragaria vesca* subsp. *vesca*	1.857
gi|470103932	9.07	3.4	3	Splicing factor 3B subunit 3-like	*Fragaria vesca* subsp. *vesca*	2.821
gi|470110556	0.08	0	1	Pentatricopeptide repeat-containing protein At1g71210-like	*Fragaria vesca* subsp. *vesca*	1.738
gi|470145892	0.14	0	1	Pentatricopeptide repeat-containing protein At2g17525	*Fragaria vesca* subsp. *vesca*	0.515
**Signal transduction**						
gi|470129261	0.32	0	1	REVERSED: rop guanine nucleotide exchange factor 1-like	*Fragaria vesca* subsp. *vesca*	6.855
gi|470112365	1.34	2.64	1	Leucine-rich repeat and death domain-containing protein 1-like	*Fragaria vesca* subsp. *vesca*	6.109
gi|470120569	0.1	0	1	REVERSED: protein tas-like	*Fragaria vesca* subsp. *vesca*	14.862
gi|470134540	0.19	0	1	REVERSED: G-type lectin S-receptor-like serine/threonine-protein kinase SD3-1-like	*Fragaria vesca* subsp. *vesca*	0.631
**Carbon metabolism**						
gi|470115774	15.61	7.15	6	Phosphoenolpyruvate carboxylase, housekeeping isozyme-like	*Fragaria vesca* subsp. *vesca*	1.888
gi|470107435	4.6	14.12	2	Isocitrate dehydrogenase [NAD] regulatory subunit 1	*NAD*	1.871
gi|470127163	7.61	10.68	3	Beta-glucosidase 44-like	*Fragaria vesca* subsp. *vesca*	0.178
**DNA metabolism**						
gi|470132957	1.85	0.71	1	FACT complex subunit SPT16-like	*Fragaria vesca* subsp. *vesca*	4.613
gi|470113538	0.1	0	1	Protein FAR1-RELATED SEQUENCE 6-like	*Fragaria vesca* subsp. *vesca*	13.428
**Transport**						
gi|470122886	0.43	0	1	Putative clathrin assembly protein At2g01600-like	*Fragaria vesca* subsp. *vesca*	10.375
gi|470130490	0.09	0	1	REVERSED: putative glycosyltransferase 5-like	*Fragaria vesca* subsp. *vesca*	9.401
gi|470135543	9	12.75	4	Probable acetyl-CoA acetyltransferase	*Fragaria vesca* subsp. *vesca*	0.581

^a^ %Cov(95) represented the ratio of detected peptides (with 95% confidence) to the protein sequence.

^b^ Peptides (95%) represented the total number of detected peptides (with 95% confidence) for the individual protein species.

With respect to the folding and degradation of proteins, we found that the levels of the chaperone ClpB1, chaperonin ClpB3, heat shock protein 90–2, heat shock cognate protein 70, heat shock protein 70, heat shock protein 22, heat shock protein 26.5, and class I heat shock protein 18.2 were up-regulated after heat treatment. The level of peptidyl cis-trans isomerase CYP38 was down-regulated (Table 2).

The chaperone proteins belong to a class of proteins associated with protein folding and degradation, and act as molecular chaperones to enhance resistance against a variety of environmental stresses. Here, we found that the amount of chaperonin ClpB3 significantly increased in response to the heat stress treatment (Table 2). The chaperones ClpB1 and ClpB3 are not only associated with protein folding under heat stress, but also participate in the reformation of thylakoid membranes of chromatophores, which can enhance the heat resistance of the chloroplast [37]. Vierling [38] found that Hsp101 (chaperone protein ClpB1) is not essential for germination and development under stress-free conditions but is necessary for heat tolerance. In a later study, Vierling [39] showed that the Arabidopsis ClpB3 protein is targeted to the chloroplast by fusing putative transit peptides and also found that its function is not restricted to heat stress, but is also essential for chloroplast development. Heat shock proteins, another class of proteins associated with folding and degradation, are widely expressed and are important for enhanced heat resistance in plants. Based on their molecular size, the heat shock proteins are divided into five categories: Hsp100, Hsp90, Hsp70, Hsp60, and sHSPs (small heat shock proteins) [28]. Hsp90s, are constitutively expressed proteins distributed in the cytoplasm or vacuole, and can regulate intracellular signal transduction through modulating protein folding to prevent protein thermal denaturation and aggregation. Xu et al. [40] reported that a wide range of signaling proteins interact with HSP90s and that these interactions are important in environmental stress responses. Hsp70s, located mainly in the cytoplasm and associated with the cytoskeleton, can assist protein refolding, directional movement of nascent proteins, and degradation of misfolded proteins [41]. Ahsan et al. [12] performed a proteomic analysis and showed that HSP70 and sHSPs are up-regulated in soybean seedlings under heat stress. sHSPs, including heat shock protein 22, heat shock protein 26.5, and the class I heat shock protein 18.2, are induced by heat stress and act as molecular chaperones to prevent protein misfolding and protein aggregation [42]. Banzet et al. [43] reported that HSP22 can protect plant cells against oxidative injury. He et al. [44] found the class I heat shock protein 18.2 is down-regulated by a cold treatment but did not show significant differential expression. In addition, small heat shock proteins are very stable, which may be helpful for self-repair mechanisms in plants following exposure to abiotic stresses [45]. In this study, the levels of heat shock protein 90–2, heat shock cognate protein 70, heat shock protein 70 and sHSPs (e.g. heat shock protein 22, heat shock protein 26.5, and class I heat shock protein 18.2) of *P*. *fruticosa* were significantly increased in *P*. *fruticosa* after 3 d at 42°C (Table 2). In summary, *P*. *fruticosa* can improve its high temperature stress response abilities by elevating the levels of particular proteins associated with synthesis, folding, and degradation.

### Proteins involved in abiotic stress and redox regulation

Glutathione reductase (GR), a translationally controlled tumor protein (TCTP) homolog, and cytochrome P450 716B2, are involved in abiotic stress resistance and redox regulation. We found that the levels of these proteins increased after heat treatment. However, the levels of thioredoxin CDSP32, and peroxide reductase Q were reduced under heat stress conditions (Table 2).

TCTP is a member of a conserved protein family and is mainly involved in the proliferation and differentiation of cells in animals [46, 47]. Research on the function of TCTP in plants is relatively sparse, although some reports suggest that *TCTP* gene expression may change under various stress conditions such as low temperature, low light, salt treatment, and water deficit [48]. Cao et al. found the expression of *BoTCTP* could be enhanced by high temperature and salt stress [49]. In our study, we found that the levels of *TCTP* homologs were up-regulated in *P*. *fruticosa* under heat stress (Table 2). This finding is similar to that reported in tomatoes treated with silicon and chitosan [50]. Overall, these results suggest that TCTP homologs are involved in stress responses in plants.

Using NADPH as the sole electron donor and reducing agent, glutathione reductase (GR) can catalyze oxidized glutathione (GSSG) and generate reduced glutathione (GSH) to maintain the balance of intracellular glutathione in plants. Moreover, GR interacts with superoxide dismutase (SOD) and ascorbate peroxidase (APX) to remove reactive oxygen species through the ascorbate-glutathione pathway [51]. Guo et al. reported that the activity of GR and SOD in corn seedlings increases when the plants are grown at 42°C [52, 53]. Here, we found that the level of GR in *P*. *fruticosa* leaves was increased under high temperature conditions (Table 1). These results show that the enhancement of gene expression and the increased protein activity of GR can help plants respond to environmental stresses such as high and low temperatures, drought, air pollution, and heavy metals.

Cytochrome P450s are the largest plant enzyme protein family and form a class of multi-function single-chain proteins that play an important function in plants [54, 55]. The P450 family is connected to secondary metabolism and participates in the biosynthesis of sterols, flavonoids, alkaloids, and terpenes to enhance protective responses in plants [56]. Thioredoxin is a highly conserved and low molecular weight protein. CDSP32 is induced by drought stress and located in the chloroplast stroma, and is abundant in young leaves [57] where it plays an important role in the maintenance of homeostasis redox states [58]. Rey et al. found that CDSP32 was more comparable to other plant thioredoxins [57]. According to previous reports, CDSP32 may catalyze BAS1 (2-Cys peroxiredoxin) by transforming into a reduced state, which can then remove peroxides and maintain the cellular homeostatic redox state [59].

Peroxide reductase is ubiquitous and can remove peroxides from plants [60]. Peroxiredoxin Q, a single molecule protein, accounts for 0.3% of the chloroplast protein, attaches to the thylakoid membrane, and is enriched in photosystem II complexes [61]. Rouhier et al. reported that expression of peroxide reductase Q in *Populus tremulus* increased following bacterial infection [60]. Kiba et al. also reported that resistance to fungi and antioxidant capacity were enhanced in transgenic maize overexpressing peroxide reductase Q homologues [62]. In summary, *P*. *fruticosa* can enhance its capacity to remove harmful substances produced by high temperature exposure and improve its resistance to high temperature stress through increasing the levels of proteins associated with abiotic stress defense and redox regulation.

### Proteins involved in photosynthesis

Photosynthesis is not only the basis of plant yield production, but is also the most sensitive physiological process to heat stress. High temperatures have a substantial effect on photosynthesis; moreover, plants need to maintain a high photosynthetic rate to ensure growth or survival at high temperatures.

In our study, we found that the levels of ferredoxin-NADP reductase, ATP synthase CF1 α subunit, 37-kDa inner envelope membrane protein, chloroplast stem-loop binding protein of 41 kDa a, and protein CHUP1 were down-regulated in plants grown under high temperature; however, levels of PRUPE_ppa002248mg and pheophorbide a oxygenase were up-regulated (Table 2).

Ferredoxin-NADP reductase and ATP synthase CF1 alpha subunit are related to photosynthetic energy metabolism. Ferredoxin-NADP reductase is mainly responsible for catalyzing the electron transfer to NADP^+^ from ferredoxin in its reduced state, and the NADPH generated is used for CO_2_ fixation in the Calvin cycle and other metabolic processes in the chloroplasts [63, 64]. Ahsan et al. also found that the levels of ferredoxin-NADP reductase are reduced when soybean leaves are exposed to 40°C for 12 h [12]. Palatnik et al. [65] found that transgenic tobacco plants expressing antisense FNR showed increased susceptibility to photo-oxidative damage. The ATP synthase CF1 α subunit is a component of ATP synthase CF1 and mainly plays a regulatory role in the synthesis or hydrolysis of ATP. Hu et al. [66] showed that that ATP synthase CF1 α chain has a lower level of expression in *Taxus wallichiana* var. *mairei* exposed to acid rain. Lee et al. [67] used a proteomic approach to show that levels of the ATP synthase CF1 α chain are increased during heat treatment at 42°C. Thus, this experiment adds evidence to the belief that the electron transport chain related to photosynthesis is affected by heat stress.

The 37-kDa inner envelope membrane protein is located in the plant chloroplast inner membrane protein, which is a functional barrier for material transport between the chloroplast stroma and cytoplasm. The chloroplast membrane protein also regulates the transport of fixed carbon produced by photosynthesis to the cytoplasm [68]. Motohashi et al. showed that the gene encoding the 37-kDa inner envelope membrane protein precursor is disrupted by a *Ds* transposon insertion in the *Arabidopsis thaliana* mutant *apg1*, and that this mutant has abnormally developed chloroplasts [69]. Therefore, the 37-kDa inner envelope membrane protein plays an important role in the development of chloroplasts in plants. The 41-kDa chloroplast stem-loop binding protein can bind to the precursors of ribosomes and participate in RNA metabolism in chloroplasts. Ariga et al. [70] found that *CSP41b* (41 kDa chloroplast stem-loop-binding protein) is significantly reduced under heat stress, which strengthens photoinhibition. The protein CHUP1 can anchor the chloroplast membrane to actin to form a bridge and thereby control the positioning of the chloroplast. Schleiff et al. found that deletion of CHUP1 function results in loss of chloroplast accumulation and avoidance response (to light) [71]. In our study, the chloroplasts in cells of *P*. *fruticosa* leaves disintegrated possibly as a consequence of the reduced level of CHUP1 during heat stress treatment.

The proteins PRUPE_ppa002248mg and pheophorbide a oxygenase are involved in the metabolism of lutein and chlorophyll, respectively. Lutein plays an important role in light damage defense, especially when plants are subjected to water, drought, and high temperature. The photosynthetic organs can reduce the excess light energy through the xanthophyll cycle pathway. Pheophorbide a oxygenase is a key enzyme in the chlorophyll catabolic pathway [72] and plays an important role in transforming pheophorbide into the primary fluorescence chlorophyll catabolite (pFCC) during the chlorophyll degradation process. Ma et al. reported that *TaPao* is involved in plant defense responses to various stresses especially in chlorophyll degradation [73]. Pheophorbide a oxygenase is positively correlated with chlorophyll degradation and the process catalyzed by this enzyme is known as green fading [74, 75], which we can directly see from our results in *P*. *fruticosa* (Fig 1D).

### Proteins involved in RNA metabolic processes

Regulation of RNA metabolism, including RNA transcription, splicing, and editing, plays an important role in plant abiotic stress responses [28]. Under high temperature stress, the levels of pentatricopeptide repeat-containing (PPR) proteins, serine/arginine-rich splicing factor RS2Z33, and splicing factor 3B subunit 3 were up-regulated (Table 2). PPRs, tandem repeat unit proteins composed of 35 degenerate amino acids, are encoded by nuclear genes. The PPR proteins mainly functions in the mitochondria or chloroplasts, but are also involved in plant growth and development, fertility restoration of cytoplasmic male sterility (CMS), the formation of organelles, RNA transcription, splicing and editing, and adversity defense [76, 77].

We found here that levels of proteins related to pre-mRNA splicing (serine/arginine-rich splicing factor RS2Z33 and splicing factor 3B subunit 3) were up-regulated in *P*. *fruticosa* leaves during high temperature stress. The serine/arginine-rich splicing factor (SR) proteins are highly conserved in eukaryotes and are necessary for fundamental and alternative splicing. Each SR member has at least one or two RNA-binding domains at the N-terminus and a dipeptide domain structure of serine and arginine (RS domain) at the C-terminal end [78–80]. SR proteins can complete the splicing of mRNA through the RNA binding domain which specifically combines with the precursor mRNA; the RS domain binds to the splicing factor allowing this factor to accumulate near the splice site of the precursor mRNA. Palusa et al. found that abiotic stresses can regulate Arabidopsis SR genes to generate surprisingly high transcriptome complexity [81]. The splicing factor 3B (SF3b) is a protein complex composed of seven protein subunits. SF3b is involved throughout the process of pre-mRNA splicing, and is important for spliceosome assembly and recognition of the intron branch point [82]. SF3b3 is an integral component of the SF3b complex and is associated with post-translational modification, protein folding, and cell morphology. Lin et al. reported that the rice regulatory factor OsSF3b3 could interact with OsSF3b2 and OsSF3b5 and mRNA splicing to mediate cell death and disease resistance [83].

### Proteins involved in signal transduction

Leucine-rich repeat and death domain-containing protein 1 (LRDD1) and Rop guanine nucleotide exchange factor 1 are involved in signal transduction and were up-regulated after heat stress treatment. The level of G-type lectin S-receptor-like serine/threonine-protein kinase SD3-1 was down-regulated (Table 2). The leucine-rich repeat and death domain-containing protein (LRDD) is a newly identified protein and contains two protein interaction domains: a death domain at the C-terminus and leucine-rich region at N-terminal region. The death domain is involved in signal transduction and apoptosis, and the leucine-rich region is involved in signal transduction and regulating reversible protein-protein interactions. Thus, our results suggest that LRDD could be a new adapter protein related to signal transduction and other cellular functions in response to abiotic stress in plants [84].

ROPs (Rho-related GTPases in plants) are important molecular switches in plant growth and development; they are involved in the regulation of cytoskeletal activity and in the generation of reactive oxygen species. They can also regulate a variety of signal transduction pathways under environmental stimuli [85, 86]. RopGEFs (Rop guanine nucleotide exchange factors) are regulatory factors that act upstream from ROPs to enable a receptor kinase to convey an extracellular signal into the cell through the conversion of GTP to GDP. In this way, the activity of ROPs mediates the transduction of intracellular information [85, 87]. Lu et al. [85] analyzed the expression of the *Arabidopsis* *thaliana* *RopGEF* family under exogenous abscisic acid (ABA) treatment and found that the expression of *RopGEFs5* is similar to that of *ROP10*, indicating that RopGEFs5 may have a regulatory function in ABA signal transduction.

The G-type lectin S-receptor-like serine/threonine-protein kinase SD3-1 is a cell surface receptor protein kinase and is important for plant growth and development, extracellular signal transduction, and disease resistance. As yet, little information is available on its response to abiotic stresses. In wild soybean, the expression of the *GsSRK* (G-type lectin S-receptor-like serine/threonine-protein kinase) gene is induced by salt, drought, and ABA [88]. Additionally, in *Arabidopsis* *thaliana*, plant height, yield, chlorophyll content, and salt tolerance of transgenic plants over-expressing *GsSRK* are higher than those of wild type plants.

### Other proteins affected during heat stress

In the high-temperature stress treatment, we also noted that the levels of some proteins involved in carbon metabolism were significantly altered, for example, DNA metabolic processes, and material transport (Table 2), isocitrate dehydrogenase [NAD] regulatory subunit 1, and phosphoenolpyruvate carboxylase. The latter two are key enzymes in the Krebs cycle and were up-regulated after high heat treatment. The FACT complex subunit SPT16-like was also increased; this protein complex affects DNA metabolic processes including replication, transcription, and repair [89]. The clathrin assembly protein, which is involved in membrane protein transportation and endocytosis, was also up-regulated in response to high temperature treatment. Overall, these differentially abundant proteins may regulate the metabolism in *P*. *fruticosa* when subjected to high temperature to improve the resistance of the plant to temperature stress.

## Conclusion

In this work, we used electron microscopy, spectrophotometry, and iTRAQ quantitative analysis techniques to characterize the changes in leaf ultrastructure, physiology, and biochemistry of *P*. *fruticosa* after a heat treatment. We also carried out functional classification and subcellular localization of proteins related to heat stress responses. The main results were: 1) the cell membrane and chloroplasts of *P*. *fruticosa* leaves are damaged by heat; 2) 58 proteins showed altered levels of abundance after heat treatment, and these proteins were involved mainly in biological processes, such as protein synthesis, folding and degradation, abiotic stress defense, photosynthesis, RNA metabolism, and signal transduction. These results improve the knowledge base for *P*. *fruticosa* thermotolerance breeding.

## Supporting information

S1 Fig Changes in the mesophyll cell ultrastructure in response to heat stress in *Potentilla fruticosa*. a, b, c and d show partial enlarged drawing of Fig 2A, 2B, 2C and 2D, respectively. Map scale: 1 μm (a, b, c, d). Ch: chloroplast; Sg: starch grain; Pg: plastoglobuli; Th: thylakoids; Mi: mitochondrion.
(TIF)

S1 Table Primers used for analysis of gene expression by qRT-PCR. (XLSX)

S2 Table Identification yields of protein, peptide at different FDR threshold by ProteinPilot. (DOCX)

S3 Table Identification of differentially abundant proteins by iTRAQ. (XLSX)

S4 Table GO annotation of differentially abundant proteins related to heat stress in *P*. *fruticosa*. (XLSX)

S5 Table The top 10 of GO enrichment of differentially abundant proteins related to heat stress in *P*. *fruticosa*. (XLSX)
