# Supplementary figures and images for: Proteomic analysis of Potentilla fruticosa L. leaves by iTRAQ reveals responses to heat stress
Source: PLoS One. 2017 Aug 22;12(8):e0182917. doi: 10.1371/journal.pone.0182917 (PMC5568749; doi:10.1371/journal.pone.0182917)

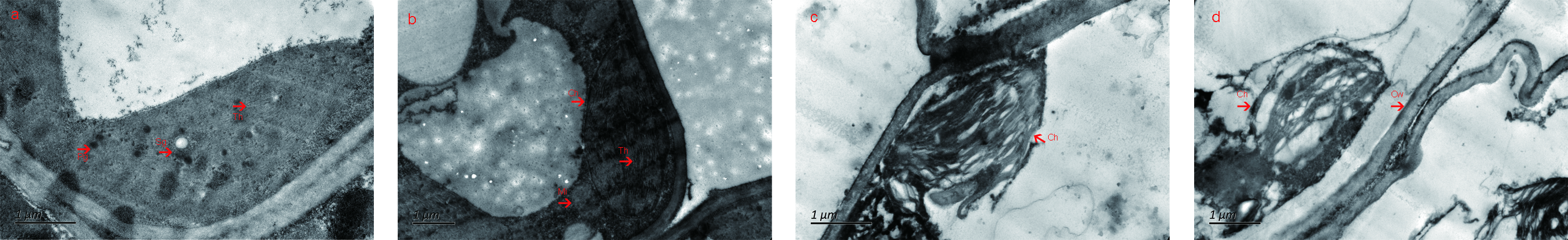

Supplement: S1 Fig — a, b, c and d show partial enlarged drawing of Fig 2A, 2B, 2C and 2D, respectively. Map scale: 1 μm (a, b, c, d). Ch: chloroplast; Sg: starch grain; Pg: plastoglobuli; Th: thylakoids; Mi: mitochondrion. (TIF) [file pone.0182917.s001.tif]
