# Supplementary material for: Proteomic analysis of Potentilla fruticosa L. leaves by iTRAQ reveals responses to heat stress
Source: PLoS One. 2017 Aug 22;12(8):e0182917. doi: 10.1371/journal.pone.0182917 (PMC5568749; doi:10.1371/journal.pone.0182917)
Supplement: S2 Table — (DOCX) [file pone.0182917.s003.docx]

**S2 Table: Identification yields of protein, peptide at different FDR threshold by ProteinPilot**

| **Data Level** | **FDR Type** | **FDR** | **ID Yield** |
| --- | --- | --- | --- |
| **Protein** | Local | 1% | *740* |
|  |  | **5%** | **800** |
|  |  | **10%** | **832** |
|  | Global | 1% | *864* |
|  |  | 5% | *942* |
|  |  | 10% | *1067* |
|  | Global FDR from Fit | **1%** | **863** |
|  |  | 5% | *968* |
|  |  | 10% | *1064* |
| **Distinct peptide** | Local | 1% | *1262* |
|  |  | **5%** | **1460** |
|  |  | **10%** | **1563** |
|  | Global | 1% | *1548* |
|  |  | 5% | *2024* |
|  |  | 10% | *2532* |
|  | Global FDR from Fit | **1%** | **1577** |
|  |  | 5% | *2017* |
|  |  | 10% | *2498* |

Note: Local FDR indicated the FDR of an individual protein, peptide and Global FDR represented the FDR applied to the entire set of proteins, peptides. FDR and ID yield in bold were the criteria for data analysis.
